# From doubt to trust: how AI safety voice bridges the gap between employees and AI teammates through perceived capability and benevolence

**DOI:** 10.3389/fpsyg.2026.1804847

**Published:** 2026-06-17

**Authors:** Zexuan Qi, Yangchun Yan, Panpan Ren

**Affiliations:** Department of Business Administration, Xinjiang College of Science and Technology, Korla, China

**Keywords:** perceived benevolence, perceived capability, safety voice, voice endorsement, willingness to work with AI

## Abstract

**Purpose:**

With the rapid advancement of artificial intelligence (AI) computing power, the creation and development of human-AI collaborative teams have become a significant trend in organizational development. However, in organizational practice, the introduction of AI is often accompanied by significant psychological resistance and algorithm aversion among employees. Based on this, this article aims to investigate the critical variable of AI safety voice, analyzing its impact mechanism on employees’ willingness to collaborate. The study focuses on examining the mediating roles of perceived AI capability and perceived AI benevolence, as well as the moderating effect of voice endorsement within this framework.

**Methods:**

Drawing upon the theory of mind perception, this study investigated mediating and moderating effects through a multi-wave questionnaire survey administered to 385 enterprise employees routinely engaged in human-AI collaboration.

**Result:**

Employees’ perceptions of AI capability and benevolence mediate the relationship between AI safety voice and employees’ willingness to work with AI. Furthermore, voice endorsement positively moderates the relationships between AI safety voice and perceived AI capability as well as perceived AI benevolence, further enhancing the indirect effect of AI safety voice on employees’ willingness to work with AI.

**Conclusion:**

The findings contribute to enhancing the relationship between human and AI teammates and provide practical insights for organizations aiming to introduce human-AI collaborative teams, thereby improving team synergy and work output.

## 1 Introduction

Artificial intelligence (AI) is demonstrating rapidly growing computational capabilities and is increasingly recognized as a collaborative teammate for humans, rather than merely a technical tool (McNeese et al., 2023). Such human-AI collaboration can enhance joint performance and tackle complex tasks that neither AI nor humans can accomplish independently (Bansal et al., 2019; Bienefeld et al., 2023). Research has demonstrated that AI can significantly enhance employee productivity (Järvelä et al., 2023), improve decision quality (Li et al., 2024), and foster creativity (Jia et al., 2024), thereby helping organizations maintain a competitive edge (Smith et al., 2025). Employees’ willingness to collaborate with AI is thus a critical factor in determining the effectiveness of these human-AI partnerships (You and Robert, 2018). Consequently, research in this field has centered on understanding human perceptions of their AI teammates and identifying strategies to foster positive perceptions and interactions, thereby encouraging this collaborative willingness (Shonhe and Min, 2025; Zercher et al., 2025).

AI demonstrates significant potential across various domains and effectively enhances workflow and decision-making efficiency. However, not all employees actively engage with AI. Existing research has found that a large proportion of employees have some degree of resistance in their interaction with AI (Quan et al., 2025). Chowdhury et al. (2022) pointed out that distrust of AI technology is an important source of employee resistance to human-AI collaboration. AI has long been seen as a tool for task execution rather than a partner involved in decision-making or driving innovation (Kang and Lou, 2022; SimanTov-Nachlieli, 2025), with employees questioning AI’s ability to tackle complex, creative or judgmental tasks (Mirbabaie et al., 2022). This suspicion of AI technology makes it difficult for employees to fully trust their role as teammates, which in turn affects their willingness to cooperate (You and Robert, 2018). Mirbabaie et al. (2022) noted that disagreement with AI teammates is an important factor in employees’ resistance to working with AI. AI is frequently stereotyped as an impersonal and unemotional tool, perceived as lacking emotional support, empathetic understanding, and adaptive social responses (Araujo, 2018; Perry, 2023). Given that employees have traditionally relied on human-centered collaboration, they tend to foster cooperation and trust through emotional resonance, nonverbal communication, and social interaction (Kang and Lou, 2022). Consequently, many employees find it difficult to perceive AI as a legitimate teammate with socio-emotional and collaborative value, which can lead to resistance to its integration into team work (De Freitas et al., 2023).

According to mind perception theory, individuals understand the characteristics of human and non-human entities through two basic dimensions: agency and experience. These dimensions influence individual responses to their attitudes and behaviors (Cuddy et al., 2008). This study suggests that AI safety voice may play a key role in enhancing employees’ willingness to work with AI. AI safety voice refers to human-AI interaction in which AI focuses on human safety and provides clear, stable, and supportive feedback aimed at providing timely safety warnings or guidance to humans in the face of potential threats or uncertain situations (Conchie, 2013; Curcuruto and Griffin, 2016). For example, AI can provide timely warnings to pilots about flight safety by monitoring flight status, weather conditions, and aircraft mechanical systems (Zaoui et al., 2024). In automated production lines, AI can monitor the operating status of equipment, detect potential equipment failures or operational errors in a timely manner, and issue safety warnings or recommendations to avoid equipment damage or personal injury (Krainiuk et al., 2023). Such safety voice enhances employees’ judgment of AI competence and perception of benevolence (Cuddy et al., 2008). Psychological perception becomes the core evaluation basis for employees to assess the value of AI collaboration, which then affects their subsequent behavioral responses (Bankins et al., 2024).

This study examines the moderating role of voice endorsement. Voice endorsement may increase employees’ willingness to work with AI by enhancing their positive perceptions of AI, such as perceived AI competence and perceived AI benevolence (Wu et al., 2024). Specifically, voice endorsement not only helps to increase employee trust in AI technology, but also encourages employees to positively evaluate AI intentions. This process may significantly increase employee willingness to work with AI by enhancing employee perceptions of competence and benevolence.

Drawing on the theory of mind perception, a moderated mediation model was developed to examine how AI safety voice affects employees’ willingness to collaborate with AI. The study specifically investigated the mediating roles of perceived AI competence and perceived AI benevolence, along with the moderating influence of voice endorsement in this process. The study makes three theoretical contributions. First, this research extends mind perception theory from traditional interpersonal interaction contexts to human-AI collaboration, systematically revealing how AI safety voice triggers employees’ dual-dimensional cognitive processes of AI capability and benevolence. Second, the study broadens the research scope of human-AI collaboration by examining the unique context of proactive AI safety voice, thereby enriching antecedent research on human employees’ psychological acceptance of AI colleagues. Third, the research expands beyond the single explanatory pathway of technology acceptance models in human-AI collaboration, offering a more comprehensive theoretical framework for understanding employees’ willingness to collaborate with AI. This integrated model provides a new theoretical perspective for the application of AI in enterprises and valuable practical insights for improving human-AI collaboration performance.

The structure of the subsequent chapters in this study is organized as follows: The second section reviews relevant literature and proposes research hypotheses. The third section outlines the methodology, covering sample selection, data collection, and variable measurement. The fourth section presents the results of the empirical analysis, including bias tests, confirmatory factor analysis, descriptive statistics, correlation analysis, and hypothesis testing. The fifth section discusses the conclusions, theoretical contributions, practical implications, limitations, and future prospects of the research findings.

## 2 Theoretical foundation and research hypotheses

### 2.1 Mind perception theory

Mind perception theory points out that individuals perceive both human and nonhuman entities through two core dimensions: agency and experience. Agency refers to the ability to control, remember, plan, and communicate, while experience refers to the ability to feel and perceive mental or physical states such as fear, pain, pleasure, and embarrassment (Gray et al., 2007). The theory further highlights the interaction between emotion and cognition in shaping individuals’ emotional and behavioral responses to external stimuli. In human-AI interaction, individuals generally attribute high agency but limited emotional experience to AI, making it challenging for employees to relate to AI in a human-like manner (K. Gray and Wegner, 2010; Waytz et al., 2010).

Mind perception theory further posits that humans attribute mental abilities to non-human entities in which they perceive human-like characteristics. For instance, robots that exhibit a high degree of personification are more likely to adhere to social norms in human interactions (Martin et al., 2020). In the context of home service robots, if a robot demonstrates self-recognition capabilities, employees may attribute certain psychological qualities to it, thereby enhancing the perceived quality of service (Söderlund, 2022). Thus, within the framework of human-AI collaboration, mind perception theory offers a unique lens to understand employee interactions with AI. Particularly when AI is perceived as a colleague rather than a mere tool, employees’ cognitive and emotional responses directly shape their cooperative attitudes toward AI. Given the lack of trust in the algorithms enclosed within the “black box,” employees, aside from evaluating AI’s technical abilities, also base their attitudes on their perceptions of AI’s intentions (Wang and Ding, 2024).

If employees hold positive views about AI’s capabilities and intentions, they are more likely to perceive AI as a trusted teammate, thereby promoting collaboration. Conversely, if employees distrust AI’s skills or perceive its behaviors as threatening, they may resist collaborating with AI. Thus, the theory of mind perception provides a crucial theoretical foundation for this study. Through this framework, the study aims to explore how perceptions of AI safety influence employees’ cognitive and emotional alignment with AI by affecting their perceptions of AI’s capabilities and benevolence, ultimately impacting their willingness to work with AI.

### 2.2 The mediating role of perceived capability

Human employees’ perception of AI capabilities, including functionality and reliability (Mcknight et al., 2011), is a crucial determinant of their attitudes and behavioral intentions toward AI technologies (Chiu et al., 2021). According to mind perception theory in human–AI collaboration, employees form perceptions of AI agency based on AI behavior, including judgments of its analysis, reasoning, and task-execution abilities (Kim and McGill, 2025). Because safety issues typically arise in emergency situations (Lainidi et al., 2023), individuals with sufficient cognitive resources are more sensitive to problem cues and more likely to detect problems before they occur (Dyne et al., 2003). AI safety voice communicates AI task effectiveness and reliability to employees by providing clear, consistent, and timely safety alerts and feedback (Chen et al., 2021). This process enables employees to positively evaluate AI’s technical capabilities and gradually build cognitive trust in AI capabilities. This competence-based trust is crucial for promoting a sense of identification with AI technology (Choung et al., 2023). Employees’ technology acceptance is closely related to their trust in AI (Chiu et al., 2021). In security-sensitive tasks, where tolerance for errors is low and consequences are serious, employees’ positive perceptions of AI capabilities are amplified, significantly enhancing their confidence in AI as a reliable partner and their willingness to collaborate with AI. Conversely, if they feel uncomfortable and insecure, they are more likely to avoid working with AI (Tu et al., 2023). Based on this, we propose the following hypothesis:

*H1*: AI safety voice positively affects employees’ willingness to work with AI by enhancing their perceived capability of AI.

### 2.3 The mediating role of perceived benevolence

Benevolence, as a key dimension of perceived emotion, encompasses traits such as altruistic behavior, compassion, and concern for the well-being of others (Chernyak-Hai et al., 2024). Mind perception theory emphasizes that an individual’s perception of nonhuman entities is not limited to their technical abilities, but also includes their recognition of human characteristics (Kim and McGill, 2025). The intention and willingness to cooperate have long underscored the importance of emotion, as humans often prioritize perceived emotion over perceived ability (Cuddy et al., 2008). Ability trust in AI technology determines whether to use AI, while emotional trust in AI technology affects the quality of collaboration, cooperation and interaction between individuals and AI (Choung et al., 2023). Existing research has found that the general consensus among humans is that AI generally has high or medium autonomy but low emotional experience. Lack of emotion is the root cause of human distrust of AI (Bienefeld et al., 2023). However, in uncertain environments, the safety voice issued by AI goes beyond merely pursuing efficiency and performance. Instead, it considers employee interests and well-being throughout the action process (Fügener et al., 2021). By conveying kind feedback such as care and support, employees can perceive AI as not only a tool, but also a teammate with good intentions. Research has revealed that when employees perceive AI’s attention to and understanding of their emotional needs, this positive experience strengthens their perception of AI’s benevolence. In uncertain environments, employees acknowledge that AI’s behavioral motivations are oriented toward protecting employee interests, which fosters employees’ emotional trust in AI and leads to greater willingness to collaborate (Bostrom et al., 2024). In addition, emotional interaction and responsiveness to employee needs help enhance employee emotional identification with AI, further facilitating collaboration (Bienefeld et al., 2023). Accordingly, we hypothesize the following:

*H2*: AI safety voice positively affects employees’ willingness to work with AI by enhancing their perceived benevolence of AI.

### 2.4 The moderating effect of voice endorsement

Voice endorsement refers to the value judgment and acceptance degree of ideas expressed by a proponent (Burris, 2012). According to the theory of mind perception, people’s behavioral responses to a target are influenced by their perceptions of both initiative and sensitivity regarding the target. These perceptions modulate the relationship between AI safety voice and perceived capability, as well as between AI safety voice and perceived benevolence.

The precise safety early warning capabilities of AI at the content quality level represent a direct expression of its autonomous functions, particularly its analytical reasoning abilities and scenario adaptation skills (Chiu et al., 2021). Whether this signal of AI capability translates into employees’ perceived capability depends on the level of voice endorsement (Lam et al., 2019). Specifically, when employees highly endorse the AI safety voice, they tend to attribute the accuracy of the warnings and the effectiveness of the feedback to the AI’s perceived technical capability, thereby strengthening the link between AI safety voice and perceived capability. Conversely, when voice endorsement is low, employees are more likely to attribute accuracy to external data completeness or coincidence, making it harder to connect AI safety voice with perceived capability and thus weakening the link between AI safety voice and perceived capability.

The AI safety voice conveys signals of benevolence, particularly as it is often expressed in polite, respectful, and supportive feedback (Conchie, 2013). The interpretation of this benevolence signal is moderated by voice endorsement (Lam et al., 2019). When employees highly endorse the safety voice, they tend to interpret the politeness and supportive feedback as expressions of AI’s active concern for employee well-being, attributing this benevolence to AI’s consideration of employee autonomy, thereby strengthening the link between AI safety voice and perceived benevolence. Conversely, when voice endorsement is low, employees are more likely to view such expressions as programmed responses of the system, which weakens the association between AI safety voice and perceived benevolence.

Thus, when employees highly endorse AI safety voice, they believe that the AI possesses professional safety capability and that its starting point is to protect employee safety. This recognition strengthens the effect of AI safety voice, leading to stronger perceived capability and perceived benevolence. Conversely, if employees do not endorse AI safety voice, they may question the AI’s expertise or motives, resulting in weaker perceived capability and perceived benevolence. Based on this, we hypothesize:

*H3*: Voice endorsement positively moderates the relationship between AI safety voice and perceived capability.

*H4*: Voice endorsement positively moderates the relationship between AI safety voice and perceived benevolence.

Based on the arguments presented above, this study argues that the influence of AI safety voice on collaboration willingness through perceived capability and perceived benevolence is contingent upon voice endorsement. Specifically, when human employees acknowledge the safety voice proposed by AI, this behavior is more readily interpreted as reflecting professional competence and benevolent intentions rather than mechanized information output. This interpretation strengthens human employees’ trust and identification with AI, ultimately enhancing their willingness to collaborate.

Low voice endorsement makes it difficult for employees to establish effective connections between AI safety voice and professional competence. This is perceived by employees as potentially being simple feedback based on preset programs rather than results from sophisticated intelligent analysis. As a result, trust in AI’s professional capabilities becomes difficult to develop (Chowdhury et al., 2022). In this state of low recognition, employees experience numerous concerns when collaborating with AI. They worry that AI may not provide reliable support in complex work scenarios, which significantly reduces their willingness to work together (You and Robert, 2018). On top of that, lower voice endorsement leads employees to perceive the safety voice issued by AI as merely following programmed instructions, lacking genuine concern for individuals in specific work situations. This creates an impression of rigidity and mechanical operation, without emotional intelligence or warmth (Li and Bitterly, 2024). Employees may feel that collaborating with such AI fails to provide the collaborative support and care, which subsequently diminishes their willingness to engage with AI technology (Tu et al., 2023).

Thus, the formation of perceived capability and perceived benevolence is closely related to voice endorsement, because endorsement provides positive feedback on the quality and emotional motives of AI safety voice. Accordingly, we propose the following moderation hypotheses:

*H5*: Voice endorsement moderates the mediating effect of perceived capability on the relationship between AI safety voice and the willingness to collaborate with AI.

*H6*: Voice endorsement moderates the mediating effect of perceived benevolence on the relationship between AI safety voice and the willingness to collaborate with AI.

**Figure 1:**
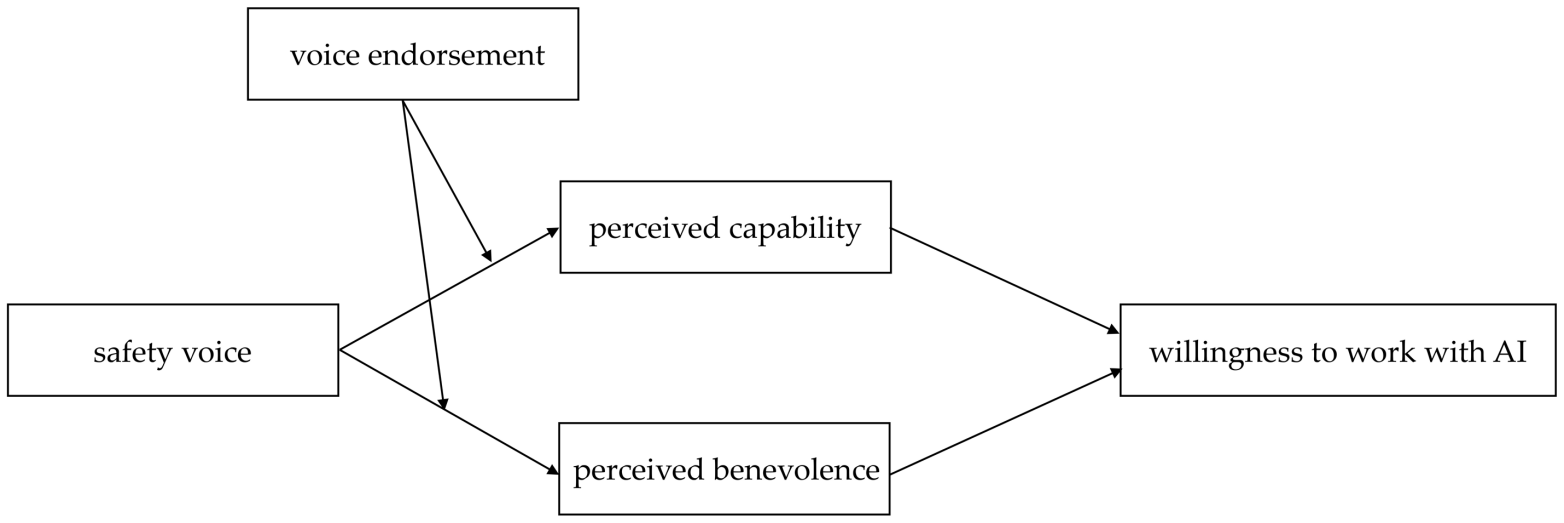
Theoretical model.

To sum up, we construct the theoretical model of this paper, as shown in Figure 1.

## 3 Methods

### 3.1 Study samples and data collection

This study employed a questionnaire survey method to collect original data, recruiting enterprise employees with experience in human-AI collaboration as participants through the Credamo online survey platform. The study utilized a non-probability sampling strategy, combining convenience and purposive sampling.

#### 3.1.1 Sampling design

Prospective participants first received a screening question: “In your past work, have you had experience with alerts, suggestions, or warnings provided by AI applications?” Those without such experience were excluded. This rigorous screening process ensured that participants possessed direct interaction experience with AI systems in a work context, particularly involving tasks related to decision support, process monitoring, or automated suggestions. This experience ensures that participants can meaningfully evaluate AI safety voice behaviors, which are particularly prominent in safety-sensitive work contexts.

#### 3.1.2 Industry and organizational context

The sample covered employees from multiple industries, including manufacturing, logistics, technology services, and energy, where AI systems are commonly embedded in operational processes. Among the final sample, 134 respondents (34.8%) worked in safety-sensitive industries (e.g., manufacturing, logistics with hazardous materials, and energy). These work contexts typically involve varying degrees of task uncertainty and potential risk, thereby increasing the practical relevance of AI safety voice in employees’ daily work experience. Furthermore, respondents were drawn from different organizational setups in which AI is implemented to varying degrees, ranging from basic AI-assisted operational support to more advanced AI-enabled decision-making systems. In these contexts, AI is used for diverse functions such as real-time monitoring, risk detection, process optimization, and decision support, reflecting heterogeneity in organizational AI adoption.

#### 3.1.3 Respondents’ AI experience

All participants reported at least 6 months of routine interaction with AI systems, with an average of 1.8 years (SD = 1.2) of human-AI collaboration experience. Additionally, 287 respondents (74.5%) reported having received at least one AI-generated safety warning or preventive suggestion in the past 6 months. Respondents also reported varying levels of experience with AI systems, from routine interaction with AI-supported tools to more intensive involvement in AI-assisted decision-making processes. This variation in experience enhances the robustness of the sample and ensures that participants possess sufficient knowledge to evaluate AI-related constructs across different organizational environments.

#### 3.1.4 Survey procedure

Before questionnaire distribution, the research objectives were clearly explained to all participants, who were instructed to respond based on their actual work experiences with AI systems. To mitigate potential common method bias, a two-wave survey design was adopted. At time point 1, participants’ demographic data (gender, age, education level, and years of work experience) and scale data regarding AI safety voice, perceived capability, and perceived benevolence were collected. At time point 2, a second questionnaire was distributed to participants who had completed the first stage, collecting data on willingness to work with AI and voice endorsement. Temporal separation between measurements helped reduce the likelihood of common method variance.

#### 3.1.5 Sample size and response rate

Economic incentives were provided to participants who successfully completed both stages of the survey to enhance response quality and participation rates. A total of 450 questionnaires were distributed. After excluding responses with substantial missing data on key measurement items, 385 valid questionnaires were retained, yielding an effective response rate of 85.6%. The final sample consisted of 31.2% male participants, with 95.1% of respondents under 50 years of age, and 4.9% having more than 15 years of professional experience. Detailed respondent information is presented in Table 1.

**Table 1:** Demographic characteristics of respondents (sample size = 385).

Characteristic	Category	Frequency	Percentage (%)
Gender	Male	120	31.2
	Female	265	68.8
Age	20–30 years	214	55.6
	31–40 years	135	35.1
	41–50 years	19	4.9
	Over 50 years	17	4.4
Education level	Associate degree or below	48	12.5
	Bachelor’s degree	276	71.7
	Master’s degree or above	61	15.8
Work experience	Less than 1 year	195	50.6
	1–5 years	105	27.3
	6–10 years	50	13.0
	11–15 years	16	4.2
	More than 15 years	19	4.9

### 3.2 Variable measurement

The scales used in this study were adapted from well-established measures in prior research. The original instruments were developed in English and translated into Chinese using a rigorous translation–back translation procedure to ensure linguistic equivalence and conceptual consistency. All constructs were measured using a five-point Likert scale ranging from 1 (“strongly disagree”) to 5 (“strongly agree”). It is important to note that the key constructs examined in this study—such as perceived capability, perceived benevolence, and willingness to work with AI—are inherently perceptual and subjective in nature. Therefore, the use of self-reported measures is appropriate and consistent with prior research in human–AI collaboration, where individuals’ interpretations of AI behavior are critical predictors of their attitudes and behavioral intentions.

#### 3.2.1 AI safety voice

The items were sourced from Tucker et al. (2008) and comprise five items, such as “AI provides suggestions to improve safety”.

#### 3.2.2 Perceived capability

The items were measured using Lv et al. (2022), comprising four items, such as “I believe AI has the necessary capabilities to carry out its work”.

#### 3.2.3 Perceived benevolence

The items were measured using Lv et al. (2022), comprising six items, such as “I believe the opinions and suggestions offered by the AI are in my best interest”.

#### 3.2.4 Willingness to work with AI

The items were sourced from You and Robert (2018), comprising two items, such as “I am willing to work with AI as part of a team”.

#### 3.2.5 Voice endorsement

The items were measured using Burris (2012), including two items such as “The recommendations provided by AI are valuable”.

#### 3.2.6 Control variables

Demographic variables, including gender, age, education, and years of service, were controlled for because prior research in technology acceptance and human–AI collaboration has shown that individual characteristics may influence employee’s perceptions of AI systems and their willingness to engage with them (You and Robert, 2018; Chiu et al., 2021; Tu et al., 2023). Specifically, age and work experience may affect individuals’ familiarity with and adaptability to new technologies, while education level may influence cognitive evaluation of AI capabilities, and gender has been found to relate to differences in technology perception in some contexts. Therefore, including these demographic variables as controls helps to isolate the effects of the focal constructs and enhances the robustness of the estimated relationships in the model.

As shown in Table 2, all standardized factor loadings exceeded 0.70, Cronbach’s alpha and composite reliability (CR) were both above 0.70, the average variance extracted (AVE) values were all greater than 0.50, and the VIF values were within the acceptable range. These results indicate that the measurement model demonstrates satisfactory internal consistency and convergent validity, with no significant multicollinearity concerns, thus providing a reliable basis for subsequent empirical testing.

**Table 2:** Summary of the measurement model.

Main constructs	Items	Items loading	VIF	Adapted from
SV	1. AI provides suggestions to improve safety.	0.810	2.369	Tucker et al. (2008)
	2. AI suggests stopping unsafe behaviors.	0.784	2.199	
α = 0.900	3. AI proposes new approaches to improve safety decision-making.	0.784	2.237	
AVE = 0.643	4. AI issues alerts when potential hazards are detected.	0.810	2.404	
CR = 0.900	5. AI issues warnings if someone violates safety regulations.	0.820	2.479	
PC	1. I believe AI has the necessary capabilities to carry out its work.	0.790	2.182	Lv et al. (2022)
α = 0.884	2. I believe AI has sufficient experience to carry out its work.	0.780	2.142	
AVE = 0.658	3. I believe AI has the resources needed to carry out its activities.	0.811	2.295	
CR = 0.885	4. I believe AI has adequate knowledge of the work and can provide us with appropriate advice.	0.861	2.701	
PB	1. I believe the opinions and suggestions offered by the AI are in my best interest.	0.764	2.158	Lv et al. (2022)
	2. I believe the AI cares about well-being.	0.808	2.409	
α = 0.910	3. I believe the AI takes into account the potential impact its actions may have on me.	0.773	2.197	
AVE = 0.629	4. I believe the AI will not do anything that harms my interests	0.818	2.546	
CR = 0.911	5. I believe the AI considers my wishes.	0.803	2.422	
	6. I believe the AI is able to understand my needs.	0.793	2.360	
WTW	1. I am willing to work with AI as part of a team.	0.878	2.314	You and Robert (2018)
α = 0.859				
AVE = 0.754	2. AI and I may become a good team.	0.859	2.314	
CR = 0.860				
VE	1. The recommendations provided by AI are valuable.	0.772	2.156	Burris (2012)
α = 0.845				
AVE = 0.748	2. I agree with the recommendations provided by AI.	0.949	2.156	
CR = 0.855				

*N* = 385; SV, safety voice; PC, perceived capability; PB, perceived benevolence; VE, voice endorsement; WTW, willingness to work with AI.

## 4 Results

### 4.1 Common method bias test

Because this study relied on self-reported data, common method bias may be a potential concern. To mitigate this issue at the design stage, several procedural remedies were implemented, including the use of well-established scales with demonstrated reliability and validity, the assurance of respondent anonymity, and the randomization of item order. These measures were intended to systematically reduce the potential influence of common method variance on the measurement results (Podsakoff et al., 2003).

To evaluate the potential for homogeneity bias, exploratory factor analysis (EFA) was conducted using Harman’s single-factor test. All questionnaire items were incorporated into the analysis, and the cumulative variance explained by the first factor was found to be 35.426%, falling below the critical threshold of 40%. These findings suggest that common method bias does not pose a significant concern in the present study, thereby permitting further analytical procedures (Podsakoff and Organ, 1986).

### 4.2 Confirmatory factor analysis

In order to verify the content validity of variables, confirmatory factor analysis (CFA) was performed using AMOS 24.0 on five variables: safety voice, perceived capability, perceived benevolence, voice endorsement and willingness to work with AI. Five-factor model fit well (*χ*^2^/d*f* = 1.024, RMSEA = 0.008, NFI = 0.967, IFI = 0.999, TLI = 0.999). See Table 3 for results. The models of safety voice combined with perceived ability (Δ*χ*^2^ = 741.418, *p* < 0.05) and safety voice combined with perceived ability and perceived kindness (Δ*χ*^2^ = 1658.171, *p* < 0.05) were significantly better than all the alternative models, and showed strong conceptual validity in the key variables studied.

**Table 3:** Results of confirmatory factor analysis.

Models		*χ*^2^/d*f*	RMSEA	NFI	IFI	TLI
1	Five-factor model	1.024	0.008	0.967	0.999	0.999
2	Four-factor model (SV × PC)	6.074	0.115	0.796	0.824	0.792
3	Three-factor model (SV × PC × PB)	12.104	0.170	0.585	0.606	0.545
4	Two-factor model (SV × PC × PB × WTW)	13.247	0.179	0.540	0.559	0.498
5	Single-factor model	15.094	0.192	0.472	0.489	0.422

*N* = 385; SV, safety voice; PC, perceived capability; PB, perceived benevolence; VE, voice endorsement; WTW, willingness to work with AI.

### 4.3 Descriptive statistics and correlation analysis

Descriptive statistics and correlations of variables are given in Table 4. There were significant positive correlations between safety voice and perceived capability (*r* = 0.333, *p* < 0.01), safety voice and perceived benevolence (*r* = 0.381, *p* < 0.01), safety voice and willingness to work with AI (*r* = 0.389, *p* < 0.01), perceived capability and willingness to work with AI (*r* = 0.445, *p* < 0.01), perceived benevolence and willingness to work with AI (*r* = 0.412, *p* < 0.01). These correlations provided preliminary support for the proposed relationships.

**Table 4:** Results of descriptive statistical analysis (*N* = 385).

Variable		Mean	S.D.	1	2	3	4	5	6	7	8
1	Gender	1.69	0.46								
2	Age	30.70	8.66	−0.005							
3	Education	2.04	0.56	0.063	−0.036						
4	Experience	1.85	1.11	0.028	0.879**	−0.061					
5	SV	3.39	0.82	−0.069	−0.060	0.014	−0.077				
6	PC	3.34	0.86	−0.041	0.005	−0.002	0.020	0.333**			
7	PB	3.31	0.80	−0.101*	−0.085	−0.094	−0.033	0.381**	0.249**		
8	WTW	3.56	0.93	−0.116*	−0.022	−0.078	0.047	0.389**	0.445**	0.412**	
9	VE	3.67	0.91	0.008	0.078	0.055	0.031	0.044	−0.066	0.078	0.020

*p < 0.05; **p < 0.01; ***p < 0.001; SV, safety voice; PC, perceived capability; PB, perceived benevolence; VE, voice endorsement; WTW, willingness to work with AI.

### 4.4 Hypothesis testing

Using Bootstrap with 5,000 subsamples, the structural model was tested at a 5% significance level. The results are presented in Table 5. Safety voice had a significant positive impact on perceived capability (*b* = 0.361, *p* < 0.001) and perceived benevolence (*b* = 0.372, *p* < 0.001). Moreover, perceived capability (*b* = 0.342, *p* < 0.001) and perceived benevolence (*b* = 0.280, *p* < 0.001) also significantly and positively influenced willingness to work with AI. These results provided foundational evidence for the mediating paths proposed in H1 and H2. Furthermore, safety voice not only had a significant indirect effect on willingness to work with AI through perceived capability (*b* = 0.124, *p* < 0.001), but also exerted a significant indirect effect on willingness to work with AI through perceived benevolence (*b* = 0.104, *p* < 0.001). These findings further supported Hypotheses H1 and H2.

**Table 5:** Test results of direct, indirect, and total effects.

Path	*b*	SE	95% CI lower	95% CI upper	*p*	Significance levels
Direct effects						
SV → PC	0.361	0.050	0.262	0.457	0.000	***
SV → PB	0.372	0.042	0.290	0.454	0.000	***
PC → WTW	0.342	0.050	0.248	0.440	0.000	***
PB → WTW	0.280	0.053	0.171	0.380	0.000	***
SV → WTW	0.222	0.051	0.122	0.324	0.000	***
VE → PC	−0.082	0.043	−0.169	0.000	0.056	
VE → PB	0.065	0.043	−0.027	0.144	0.136	
SV × VE → PC	0.205	0.049	0.113	0.305	0.000	***
SV × VE → PB	0.244	0.051	0.148	0.344	0.000	***
Indirect, and total effects						
SV → PC → WTW	0.124	0.025	0.082	0.182	0.000	***
SV → PB → WTW	0.104	0.024	0.061	0.157	0.000	***
Total indirect (point *M*)	0.228	—	—	—	—	
Total effects (point *M*)	0.450	0.055	0.348	0.559	0.000	***

Indirect and total effects were estimated at the mean level of the moderating variable (*M* = 0); CI was a 95% bias-corrected bootstrap. SV, safety voice; PC, perceived capability; PB, perceived benevolence; VE, voice endorsement; WTW, willingness to work with AI. * *p* < 0.05; ** *p* < 0.01; *** *p* < 0.001.

**Figure 2:**
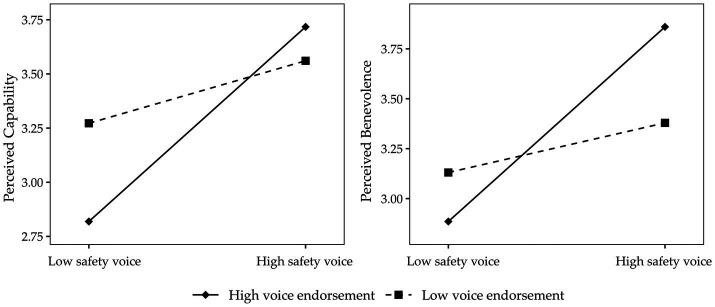
The moderating role of voice endorsement.

The moderating effect of voice endorsement on the relationships between safety voice and perceived capability and perceived benevolence was further examined. The interaction term between safety voice and voice endorsement (SV × VE) had a significant positive effect on perceived capability (*b* = 0.205, *p* < 0.001). In addition, the interaction term between safety voice and voice endorsement (SV × VE) also had a significant positive effect on perceived benevolence (*b* = 0.244, *p* < 0.001), as shown in Table 5. To clearly illustrate the effect of the interaction between safety voice and voice endorsement on the mediating variables, simple slope analyses were conducted at one standard deviation above and below the mean (+1SD/−1SD), as shown in Table 6 and Figure 2. When voice endorsement was high (+1SD), the effect of safety voice on perceived capability was very strong (*b* = 0.547, *p* < 0.001), whereas when voice endorsement was low (−1SD), this effect was significantly reduced (*b* = 0.175, *p* < 0.05). Similarly, when voice endorsement is high (+1SD), the positive relationship between safety voice and perceived benevolence is stronger (*b* = 0.593, *p* < 0.001), whereas when voice endorsement is low (−1SD), this positive effect decreases (*b* = 0.152, *p* < 0.05). Therefore, H3 and H4 are supported.

**Table 6:** Simple slope analysis.

Path	Moderating variable	*b*	SE	95% CI lower	95% CI upper	*p*	Significance levels
SV → PC	Low VE (*M* − 1SD)	0.175	0.073	0.018	0.306	0.017	*
SV → PC	High VE (*M* + 1SD)	0.547	0.060	0.428	0.661	0.000	***
SV → PC	Difference (high − low)	0.372	0.088	0.205	0.551	0.000	***
SV → PB	Low VE (*M* − 1SD)	0.152	0.073	0.006	0.289	0.038	*
SV → PB	High VE (*M* + 1SD)	0.593	0.050	0.488	0.688	0.000	***
SV → PB	Difference (high − low)	0.442	0.093	0.268	0.623	0.000	***

*b* denotes the unstandardized simple slope, and SE and the 95% CI are obtained from the Mplus bootstrap procedure (5,000 replications, bias-corrected). SV, safety voice; PC, perceived capability; PB, perceived benevolence; VE, voice endorsement. * *p* < 0.05; ** *p* < 0.01; *** *p* < 0.001.

To further examine the moderated mediating effect, a Bootstrap analysis with 5,000 resampled samples was conducted to test the conditional indirect effects at ±1 standard deviation (SD) of the moderating variable. The results are presented in Table 7. The results revealed that the mediating effect of safe voice on willingness to work with AI through perceived capability gradually strengthened as the level of the moderating variable, voice endorsement, increased. This effect increased from a low level (*b* = 0.060, *p* < 0.05) to a moderate level (*b* = 0.124, *p* < 0.001), and then to a high level (*b* = 0.187, *p* < 0.001). Similarly, the mediating effect of safe voice on willingness to work with AI through perceived benevolence also gradually strengthened as the level of the moderating variable, voice endorsement, increased. This effect increased from a low level (*b* = 0.042, *p* = 0.075) to a moderate level (*b* = 0.104, *p* < 0.001), and then to a high level (*b* = 0.166, *p* < 0.001). Therefore, H5 and H6 are supported.

**Table 7:** Conditional indirect effect test results.

Path	Moderating variable	*b*	SE	95% CI lower	95% CI upper	*p*	Significance levels
SV → PC → WTW	Low VE (*M* − 1SD)	0.060	0.028	0.009	0.119	0.032	*
	Mean VE (*M*)	0.124	0.025	0.082	0.182	0.000	***
	High VE (*M* + 1SD)	0.187	0.031	0.134	0.257	0.000	***
	Difference (high − low)	0.127	0.031	0.076	0.200	0.000	***
SV → PB → WTW	Low VE (*M* − 1SD)	0.042	0.024	0.003	0.099	0.075	
	Mean VE (*M*)	0.104	0.024	0.061	0.157	0.000	***
	High VE (*M* + 1SD)	0.166	0.033	0.103	0.229	0.000	***
	Difference (high − low)	0.124	0.032	0.070	0.195	0.000	***

CI was a 95% bias-corrected bootstrap (5,000 replications). SV, safety voice; PC, perceived capability; PB, perceived benevolence; VE, voice endorsement; WTW, willingness to work with AI. * *p* < 0.05; ** *p* < 0.01; *** *p* < 0.001.

## 5 Discussion and conclusions

### 5.1 Theoretical integration and interpretation

Drawing on mind perception theory, this study provides a theoretical explanation for how employees interpret and respond to AI behaviors in collaborative contexts. Specifically, the findings suggest that AI safety voice functions as a critical behavioral signal that activates employees’ cognitive and affective evaluations of AI, thereby shaping their willingness to collaborate. From the perspective of mind perception theory, individuals evaluate non-human agents along two fundamental dimensions: agency and experience. In the context of human–AI collaboration, AI safety voice serves as a salient cue that conveys both functional competence and intentional benevolence. This dual signaling mechanism explains why AI safety voice can simultaneously enhance employees’ perceived capability and perceived benevolence of AI. More importantly, this study extends existing theoretical understanding by demonstrating that employees’ responses to AI are not solely driven by technical performance, but also by their interpretation of AI’s intentions and social meaning. The results show that when AI provides safety-related feedback, employees are more likely to attribute both expertise and prosocial motives to AI, thereby reducing uncertainty and increasing their willingness to work with AI.

In addition, the moderating role of voice endorsement further enriches the theoretical model. Voice endorsement reflects employees’ evaluative judgment of AI-generated suggestions and determines whether AI safety voice is interpreted as meaningful and credible. When endorsement is high, employees are more likely to internalize AI signals as indicators of competence and goodwill, thereby strengthening the effects of AI safety voice on both perceived capability and perceived benevolence. Conversely, when endorsement is low, AI safety voice is more likely to be perceived as routine or mechanical output, weakening its psychological impact.

Overall, these findings highlight a key theoretical mechanism: AI behavioral signals → human perceptual interpretation → collaboration willingness, which bridges mind perception theory and human–AI interaction research.

### 5.2 Theoretical significance

First, this study employs the theory of mind perception as an explanatory framework to reveal how AI safety voice triggers employees’ mental cognition of AI across two dimensions: perceived capability and perceived benevolence. This theory is extended from traditional interpersonal interaction contexts to the domain of human-AI collaboration. Traditional theory of mind perception focuses on how humans infer others’ mental states through the dual dimensions of agency and experience. This study validates the applicability of this theory to non-human entities. The agency and experience perspectives reveal the key role of human-machine cooperation behavioral transmission mechanisms in explaining the transformation of AI behavior into human social attributes. AI is endowed with technological agency and emotional experience. The essence of trust construction in human-machine collaboration is revealed to be the projection of social cognitive processes, providing a new perspective for understanding the cognition of human-machine trust. This finding not only confirms that mind perception theory can be applied to non-human agents (Chen et al., 2024), but also uncovers the cognitive mechanisms underlying anthropomorphic mentalization in human-AI interactions (Yin et al., 2024).

Second, this study expands the scope of human-AI collaboration research and enriches antecedent studies on human employees’ acceptance of AI colleagues. Existing literature finds that most users do not take a firm stand when accepting or rejecting AI, and in tasks, user support and opposition to AI may exist simultaneously and interact (Quan et al., 2025). This study reveals the unique value of safety-oriented technology behavior in building human-AI trust, confirms the relationship between AI safety voice and employees’ willingness to work with AI, and proposes that AI safety voice, as a special behavior with technical functionality and social responsibility, can activate employees’ deep trust in AI. Compared with AI’s conventional technical behavior, safety voice is directly related to human core interests, and its essence is that AI actively participates in early warning and avoidance of potential threats. This protection of employees’ fundamental interests makes it easier for employees to accept AI as a working partner by satisfying their personal utility such as safety and security (Park et al., 2024).

Third, it expands the single explanatory path of the technology acceptance model in human-AI collaboration. The study found parallel mediation mechanisms of perceived AI capability and perceived AI benevolence on employees’ willingness to work with AI, extending the limitations of the traditional Technology Acceptance Model (TAM) that focuses on performance utility such as perceived usefulness and perceived ease of use. This conclusion extends the traditional assumption of instrumental rationality in human-AI collaboration research and demonstrates that affective cognition can function independently of technical effectiveness (Gelbrich et al., 2021; Schiavo et al., 2024). The research provides a bridge for integrating technology acceptance theory and organizational trust theory, emphasizes that human-AI collaboration needs to meet the double standards of technical effectiveness and social goodwill at the same time, and lays a theoretical foundation for exploring other emotional driving mechanisms in the future.

### 5.3 Management insights

Building on the above theoretical insights, this study offers several practical implications for organizations implementing AI systems.

First, our findings indicate that the safety voice offered by artificial intelligence can enhance employees’ perceptions of its ability and benevolence. Therefore, organizations should prioritize optimizing how such advice is formulated and presented when designing AI interactive systems. Specifically, they need to refine the design of AI suggestions. These should be supported by accurate and objective data to substantiate professional judgments and framed in a collaborative and humanized manner to convey supportive intent. This approach can reduce employees’ doubts about AI’s capabilities and mitigate defensive reactions. Additionally, organizations should establish a risk-oriented, hierarchical triggering mechanism. Proactive safety interventions should be strictly limited to medium or high risk scenarios to avoid negative employee emotions that may arise from diminished autonomy or excessive prompting.

Second, managers need to institutionalize measures to strengthen employees’ recognition of the value of AI safety voice. Organizations should establish a tracking mechanism for the effect of safety voice, when employees adopt the recommendations and avoid risks, timely and automatically generated through the system performance report or associated safety performance appraisal of its value, objective data to verify the reliability of the AI capabilities and enhance the perception of the effect of the employees on the advice. At the same time, the organization should set up human-AI collaboration dispute handling process, open a multi-departmental joint assessment channel for employees questioning AI suggestions to ensure the objectivity of the conclusions, in order to reduce employee resistance due to misunderstandings, and to maintain the impartiality of the organization’s decision-making.

Third, organizations need to reduce understanding bias in human-AI collaboration through role-specific training. For employees, training should focus on explaining the behavioral logic of AI safety voice, helping employees rationally understand the motivation of AI decision-making, so as to reduce the negative emotions caused by misunderstanding and enhance the recognition of the value of AI collaboration. For managers, the training should focus on human-AI conflict management and safety performance communication skills to enhance their ability to promote human-computer mutual trust, so as to fully realize the positive impact of managerial recognition on employee acceptance of AI.

### 5.4 Conclusion

Drawing on the theory of mind perception, this study examines how AI safety voice influences employees’ willingness to work with AI. The research investigates the mediating roles of perceived AI capability and perceived AI benevolence, along with the moderating effect of voice endorsement. Data were collected at multiple time points, and the findings reveal:

First, AI safety voice positively affects employees’ willingness to collaborate with AI through their perceptions of AI capability and AI benevolence. When AI demonstrates professional safety capabilities, it strengthens employees’ confidence in its technical reliability and reduces concerns about the unpredictability of AI recommendations. Meanwhile, perceived AI benevolence helps bridge emotional gaps in human-AI collaboration, encouraging employees to view AI as a cooperative partner with social intelligence. These dual pathways of capability and benevolence perception decrease employees’ sensitivity to collaboration risks and foster their inclination to work alongside AI.

Second, voice endorsement positively moderates the relationships between AI safety voice and both perceived AI competence and perceived AI benevolence. Additionally, voice endorsement positively moderates the relationship between AI safety voice and employees’ willingness to collaborate with AI. When employees provide substantive endorsement of AI voice behaviors, they tend to interpret AI safety behaviors as effective and genuine collaborative signals. Such endorsement strengthens the perceived professionalism of AI competence while emphasizing the trustworthiness of AI’s benevolent intentions. The findings further reveal that in high voice endorsement contexts, AI safety voice demonstrates a more pronounced positive effect on employees’ willingness to work collaboratively.

## 6 Limitations

This study has several limitations that should be acknowledged. First, although a two-wave questionnaire design was adopted to mitigate common method bias, the data were collected at only two time points and thus cannot fully capture the dynamic evolution of the focal variables over time. Future research could employ longitudinal or panel designs with multiple measurement waves to more precisely examine the temporal dynamics underlying human–AI collaboration processes. Second, the study relies on self-reported data, which may introduce subjective biases. However, it is important to note that the core constructs examined in this research—such as perceived capability, perceived benevolence, and willingness to work with AI—are inherently perceptual in nature and therefore appropriately measured through self-report instruments. Nevertheless, future research could complement perceptual data with objective indicators (e.g., behavioral outcomes, system logs, or performance metrics) or adopt experimental designs to further strengthen causal inference and enhance robustness. Third, although participants were required to have prior experience with AI systems in work contexts, the study does not fully differentiate between varying levels of AI expertise, usage intensity, or familiarity with different types of AI applications. These differences may influence individuals’ ability to accurately evaluate AI behaviors, particularly in complex or high-stakes scenarios. Future research could incorporate more fine-grained measures of AI experience and expertise to better capture heterogeneity among respondents. Fourth, while this study focuses on AI safety voice, it does not explicitly distinguish between high-risk and low-risk work environments. Given that safety voice is likely to be more salient and consequential in safety-critical contexts (e.g., manufacturing, healthcare, or aviation), the generalizability of the findings across different levels of task risk may be limited. Future research could explicitly compare samples across varying risk contexts to examine whether the proposed relationships differ in safety-critical versus non-critical environments. Finally, this study examines the mechanisms at the individual level based on mind perception theory. Future research could adopt a cross-level perspective to explore how organizational-level factors—such as AI implementation maturity, organizational safety climate, or leadership support for AI—shape or moderate the relationships identified in this study. Such an approach would further enrich the explanatory power and contextual applicability of the theoretical framework.

## Data Availability

The original contributions presented in the study are included in the article/supplementary material, further inquiries can be directed to the corresponding author.
